# Oxidative Stress in Retinal Degeneration Promoted by Constant LED Light

**DOI:** 10.3389/fncel.2019.00139

**Published:** 2019-04-11

**Authors:** Maria M. Benedetto, Maria A. Contin

**Affiliations:** ^1^Departamento de Química Biológica Ranwel Caputto, Facultad de Ciencias Químicas, Universidad Nacional de Córdoba, Córdoba, Argentina; ^2^Centro de Investigaciones en Química Biológica de Córdoba (CIQUIBIC), CONICET, Universidad Nacional de Córdoba, Córdoba, Argentina

**Keywords:** retinal light damage, LED light, oxidative stress, fatty acid, electroretinogram

## Abstract

Light pollution by artificial light, might accelerate retinal diseases and circadian asynchrony. The excess of light exposure is a growing problem in societies, so studies on the consequences of long-term exposure to low levels of light are needed to determine the effects on vision. The possibility to understand the molecular mechanisms of light damage will contribute to the knowledge about visual disorders related to defects in the phototransduction. Several animal models have been used to study retinal degeneration (RD) by light; however, some important aspects remain to be established. Previously, we demonstrated that cool white treatment of 200 lux light-emitting diode (LED) induces retinal transformation with rods and cones cell death and significant changes in opsin expression in the inner nuclear layer (INL) and ganglion cell layer (GCL). Therefore, to further develop describing the molecular pathways of RD, we have examined here the oxidative stress and the fatty acid composition in rat retinas maintained at constant light. We demonstrated the existence of oxidative reactions after 5 days in outer nuclear layer (ONL), corresponding to classical photoreceptors; catalase (CAT) enzyme activity did not show significant differences in all times studied and the fatty acid study showed that docosahexaenoic acid decreased after 4 days. Remarkably, the docosahexaenoic acid diminution showed a correlation with the rise in stearic acid indicating a possible association between them. We assumed that the reduction in docosahexaenoic acid may be affected by the oxidative stress in photoreceptors outer segment which in turn affects the stearic acid composition with consequences in the membrane properties. All these miss-regulation affects the photoreceptor survival through unknown mechanisms involved. We consider that oxidative stress might be one of the pathways implicated in RD promoted by light.

## Introduction

The disturbance between the amount of reactive oxygen species (ROS) and antioxidants production is defined as oxidative stress. This imbalance produces tissue injury (Halliwell, 1994). The retina carries out the capture of light photons, and for this, it is exposed to suffer oxidative stress. However, it has many mechanisms to counteract these processes through the action of antioxidant enzymes as superoxide dismutase (SOD), catalase (CAT) and glutathione peroxidase (GSHPx) and vitamins as ascorbic acid, vitamin E, melanin (Iusifov et al., 1980; Scibior and Czeczot, 2006; Pavarino et al., 2013). The overexposure to light may be one of the many factors that can induce the interruption of this homeostasis, promoting the injury of eye tissues, cell death or stimulating simultaneously antioxidant protection by up-regulation of antioxidant enzymes (Yusifov et al., 2000); however, when the equilibrium is broken the consequence is the induction of retinal degeneration (RD). In mammals, in normal light conditions, the retina fulfills two main roles: the vision over rods and cones activity and the non-image forming tasks including circadian entrainment, pupillary response to light, secretion of melatonin and sleep regulation (Berson et al., 2002; Rollag et al., 2003; Guido et al., 2010; Sikka et al., 2014). Light stimulates photoreceptor cells activating the phototransduction cascade which promotes hyperpolarization (Sung and Chuang, 2010). Light overexposure-induced RD might induce retinal task deficits with consequences in the secretion of melatonin, desynchronization of rhythms such as sleep/wake, among others. The use of different sources of artificial light is increasing in actual society but this promotes changes in human behavior. Furthermore, the use of technologies such as light-emitting diode (LED), smart TVs and cell-phones, promote an excess of blue light exposure especially at night. Albeit the effects of artificial illumination are unknown, it may have a strong impact on retinal functions with negative consequences on people’s health. Considering that the excess of light exposure constitutes an upcoming polluter, it is clearly an emerging public health issue. Different animal models have been used by researchers in order to study the processes of RD promoted by light exposure. Even though Noell et al. (1966) suggested that exposure to light produces retinal changes, this phenomenon has not been fully clarified yet (Shear et al., 1973; Rapp and Williams, 1979; Sperling et al., 1980; Semple-Rowland and Dawson, 1987; Remé et al., 2000; Organisciak and Vaughan, 2010; Roehlecke et al., 2013; Shang et al., 2014). In retina and retinal pigment epithelium (RPE) cells, the exposure to blue light inhibits the mitochondrial enzymes and cytochrome oxidase expression inducing retinal damage (Chen, 1993; Cai et al., 2000; Roehlecke et al., 2011) suggesting the existence of oxidative stress mechanisms as part of the retinal cell death mechanism induced by light exposure. Recently, Nakamura et al. (2017) established that LED light exposure during 2 h (800 lx) induced retinal damage where oxidative stress was partially involved. However, the consequences of constant low light exposure in which the phototransduction mechanism is constantly activated for long time remain unknown. In this regard, we have established and characterized a model of RD induced by a constant exposure to LED. We have showed that classical photoreceptor cell death without caspase-3 activation and a gradual increase in levels of rhodopsin-phospho-Ser334 as a result of light exposure, suggesting that constant light produces changes in the regulation of phototransduction in rods (Contín et al., 2013).

However, retinal ganglion cells (RGCs) do not die after constant light treatment (LL), suggesting a neuroprotection mechanism also involved in our model (Benedetto et al., 2017). So, the key questions in the present work are: does the overexposure at constant low levels of LED light yield oxidative stress? What is the kinetic of oxidative stress production if the exposure is constant? Therefore, the goals of this work were to investigate the existence of oxidative stress, fatty acid composition and retinal function during the different days of LL stimulation in retinas of Wistar rats.

## Materials and Methods

### Animals

All animal procedures were performed in accordance with the protocol approved by the local animal committee (School of Chemistry, UNC, Exp. 2018-740), in accordance with the ARVO statement for the use of animals in ophthalmic and vision research. Wistar rats from 12 to 15 weeks of age were maintained with food and water *ad libitum* and illumination cycle from 12:12 h (light/dark) with white fluorescent light on (~50 lux) from Zeitgeber time (ZT) 0–12 from the time they were born, up to the experiment.

### Light Damage

#### Constant Light

RD was induced as described by Contín et al. (2013). Briefly, rats were exposed to constant light in boxes with LED lamps (EVERLIGHT Electronic Co., Ltd. T-13/4 3294-15/T2C9-1HMB, color temperature of 5,500 K) in the inner upper surface and temperature-controlled at 24 ± 1°C. At the level of the rats’ eyes, 200 lx were measured with a light meter (model 401036; Extech Instruments Corp., Waltham, MA, USA). After 1 to 8 days of constant light stimulation (LL1–LL8) the animals were killed in a CO_2_ chamber at ZT6. Controls in light dark cycle (LD), with LED or fluorescent light (RT) and constant darkness (DD) were exposed for 7 days.

#### Dark Period Protocol

Animals were subjected to a constant light during 8 days with periods of dark during 2, 4, 6, 10 and 12 h every day at the beginning of the subjective night (ZT6) under identical conditions as animals exposed to LL, in the temperature-controlled stress box at 24 ± 1°C.

### Electroretinograms (ERGs)

The methods employed for Scotopic electroretinogram (ERG) were as previously described by Dorfman et al. (2014) using an ERG machine (Akonic BIOPC, Buenos Aires, Argentina). Briefly, first animals were adapted to dark for 20 min. Then, they were anesthetized with an intraperitoneal injection containing a solution of xylazine hydrochloride (2 mg/kg) and ketamine hydrochloride (150 mg/kg). Pupils were dilated with tropicamide (1% Alcon Laboratories) and, in order to prevent eye dehydration and permit electrical contact activity when the electrode is recording, the cornea was irrigated with proparacaine hydrochloride (0.5% Alcon Laboratories). Both eyes were recorded simultaneously applying flashes of white light (5 ms, 0.1 Hz) from a photostimulator set at maximum brightness (3 cd s/m^2^ without filter). Then, the recordings were amplified and filtered (1.5 Hz low-pass filter, 300 Hz high-pass filter, notch filter activated). An average of 10 responses for each eye was measured. Mean “a” and “b” waves peak latencies and amplitudes of the responses from each group of rats were compared.

### Outer Nuclear Layer Analysis

The retinal fixation method, sectioning and nuclear quantification were as previously described (Contín et al., 2013). Briefly, rats’ eyes were fixed overnight at 4°C in 4% (W/V) paraformaldehyde in 100 mM sodium phosphate buffer (PBS, pH 7.3). Then, they were cryoprotected in sucrose and mounted in an optimal cutting temperature compound (OCT; Tissue-Tek^®^ Sakura). Retinal sections were cut along the horizontal meridian (nasal-temporal). The sections were stained with 1% Hoechst (33258 Sigma Aldrich) for 5 min and photographed using a confocal microscope (Olympus FV1200, Japan) at 40× magnification. The nuclei were counted in “left, middle left, middle right and right” designated areas from five different animals per treatment using the software ImageJ (v. 1.45) and the plugin “Automatic Nuclei Counter”.

### Superoxide Production

Dihydroethidium [(DHE; sigma 37291-25 mg dihydroethidium at 10-mg/mL stock solution in dimethylsulfoxide)], a redox-sensitive probe, was used to detect superoxide generation as previously described (Peng et al., 2011). Briefly, eyes were removed, washed in PBS solution and then incubated with 1 mM DHE for 12 h at room temperature in PBS. Then the eyes were incubated in paraformaldehyde 4% for 12 h at room temperature, harvested and quikly frozen in liquid nitrogen for cryosection (Leica CM 1950, Leica Microsystems Ltd, Wetzlar, Germany). The cryosections (10 μm) were analyzed by confocal microscopy (Olympus FV300, Japan). The DHE molecules enter the cells and are oxidized by superoxide contained inside cells, to ethidium (Et) which is fluorescent. This product is retained in the cell allowing the estimation of cellular superoxide production.

### Oxidative Stress Quantification

ROS molecules were determined by flow cytometry with 2′,7′-dichlorofluorescein diacetate (DCFH-DA) probe (D6883 Sigma). DCFH-DA is sensitive to oxidation and is nonfluorescent, it may be oxidized by ROS and peroxides and become a fluorescent molecule, DCFH (Gomes et al., 2005). Briefly, retinas dissected and dissociated in 0.25% (W/V) trypsin (Life Technology, Inc., Carlsbad, CA, USA) for 7 min at 37°C were centrifuged at 3,000 rpm for 3 min, incubated with 200 μl of trypsin inhibitor (STI) for 3 min and washed twice with PBS 1×. Then, they were suspended in 200 μl of PBS 1×, incubated with DCFH-DA (5 μM) for 60 min at 37°C in dark, washed twice with PBS 1× and analyzed on a Becton-Dickinson FACS flow cytometer; the excitation and emission wavelengths were set at 488 and 525 nm, respectively. Data were analyzed with FlowJo software (LC, Ashland, Ore). Three independent experiments were performed and the results were expressed as mean ± standard error (SE) in arbitrary units of DCFH fluorescence intensity.

### Catalase Activity Detection

Catalase (CAT) activity was assessed according to Aebi (1984). Briefly, individual retinas were homogenized in PBS 1×. Then, 1.3 ml of Buffer Phosphate (50 mM, pH 7.4) and 1.25 ml of distilled H_2_O were added to 300 μl of previously homogenized sample. Subsequently, 150 μl of H_2_O_2_ (300 mM) was added and the spectrophotometric changes were detected by measuring the absorbance at 240 nm for 1 min. The protein concentration was determined by Bradford (1976).

### Lipid Extraction From Rat Retinas

Lipids were extracted from the samples as Folch’ method slightly modified (Folch et al., 1957). Briefly, collected retinas were homogenized in 200 μl of MiliQ H_2_O and 3 ml of a combination of Chloroform: methanol [(2:1; v/v)] and 600 μl of MiliQ H_2_O were added and vortexed vigorously for 5 min and centrifuged at 2,000 rpm at 15°C for 10 min. The layer containing chloroform was collected and 1.5 ml of a mixture of chloroform: methanol: H_2_O [(3:48:47; v/v/v)] was added, mixed gently and centrifuged 10 min at 2,000 rpm. Finally, chloroform was dried under a stream of N_2_ and the extracts stored for further experiments.

#### Fatty Acid Methyl Esters Analysis by Gas Chromatography/Mass Spectrometry With Ion-Trap Detector

Extracted lipids were transmethylated to fatty acid methyl esters (FAMEs) with sodium methoxide and toluene [(2:1; v/v)] overnight at 4°C. FAMEs were then extracted with hexane and analyzed by using a GC/MS (Saturn^®^ 2000 GC/MS, Agilent, Santa Clara, USA) equipped with an ion-trap detector and a fused capillary column (HP-5MS, 30 mm × 0.25 mm i.d × 0.25 μm film thickness, Agilent, Santa Clara, USA). The carrier gas used was Helium (flow rate 1.0 ml/min); the detector and injector temperature were maintained at 150°C and 300°C, respectively. The injection (split-splitless injector) volume of the sample was 1 μl with a split ratio of 1:50. The oven was programmed as follows: 170°C for 3 min—200°C at the rate of 1.2°C/min—10°C/min to 240°C—80°C/min to 280°C and 80°C/min to 300°C with a final hold for 46.7 min.

The column temperature was programmed as follows: 170°C; 1.2°/min to 200°C—10°/min to 240°C—80°/min to 2,800°C—80°/min to 300°C and hold at 300°C for 4 min. Then, FAMEs were identified comparing mass spectra with dates in the library (National Institute of Standards and Technology, Gaithersburg, MD, USA). Furthermore, we compared FAMEs retention times with the commercial standard Supelco 37-Component FAME Mix (Sigma Aldrich, St. Louis, MO, USA).

### Statistical Analysis

Statistical analysis was carried out using the Infostat software (Version 2017, InfoStat Group, FCA, National University of Córdoba, Argentina). One-way analysis of variance (ANOVA) was used for statistical comparisons. In the text and figure, data are expressed as mean ± SD or SE. It was considered statistically significant a *p*-value < 0.05. The normality and homogeneity of the variance assumptions were proved with Shapiro–Wilks and Levene tests, respectively. Duncan *post hoc* test with a *p* value < 0.05 were considered statistically significant. A non-parametric Kruskal-Wallis test was performed when the data did not comply with the assumptions of the ANOVA. Spearman correlation coefficients were calculated to determine associations between the content of fatty acids.

## Results

### Structural Retinal Analysis

**Figure 1:**
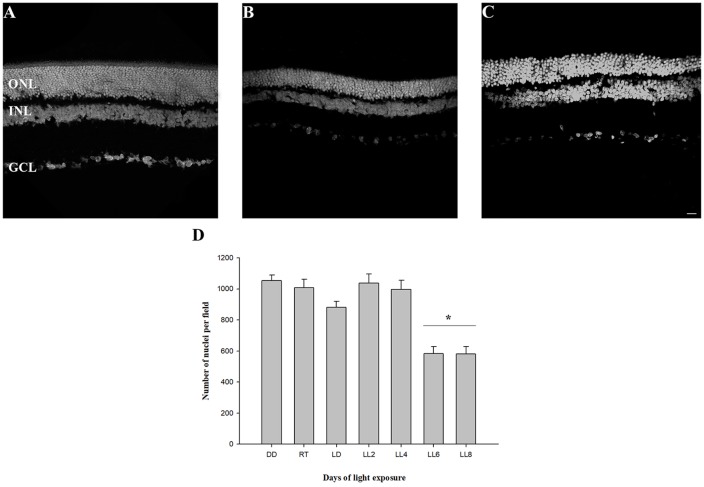
Outer nuclear layer analysis. **(A)** Rat retinas maintained in dark for 7 days (DD). **(B–C)** Retinas from rats exposed 6 (LL6) or 8 (LL8) days to constant light (200 lx). **(D)** Quantification of ONL nuclei; rat retina images corresponding to controls [(LD cycle, RT cycle and non-exposure to light (DD)] or exposure to 200 lx LED light (LL) for 2, 4, 6, and 8 days were analyzed to quantify photoreceptor survival. Data are mean ± standard error (SE), *n* = 2 animals/group from five independent experiments, *p < 0.05 vs. DD and RT by one-way analysis of variance (ANOVA) and Duncan’s *post hoc* test. ONL, outer nuclear layer; INL, inner nuclear layer; GCL, ganglion cell layer. Scale bar indicates 30 μm.

In order to know the kinetic of cell death events we analyzed the retinal layers, oxidative stress, CAT activity, fatty acid composition and ERG activity in animals exposed to constant light. The analysis of retinal structure revealed a reduction in outer nuclear layer (ONL) thickness at LL6 and LL8 compared with animals reared in DD (Figures 1A–C). The quantification of nuclei in this area showed a significant reduction after 6 and 8 days of constant light (583.04 ± 45.31 and 580.10 ± 45.31 nuclei, respectively) compared with DD and RT [(1052.19 ± 38.41 and 1007.63 ± 54.32 nuclei, respectively, *p* < 0.005, Figure 1D)]; indicating that between LL0 to LL6 are being carried out the photoreceptor cell death pathways.

### Oxidative Stress Study

#### DHE Determination

**Figure 2:**
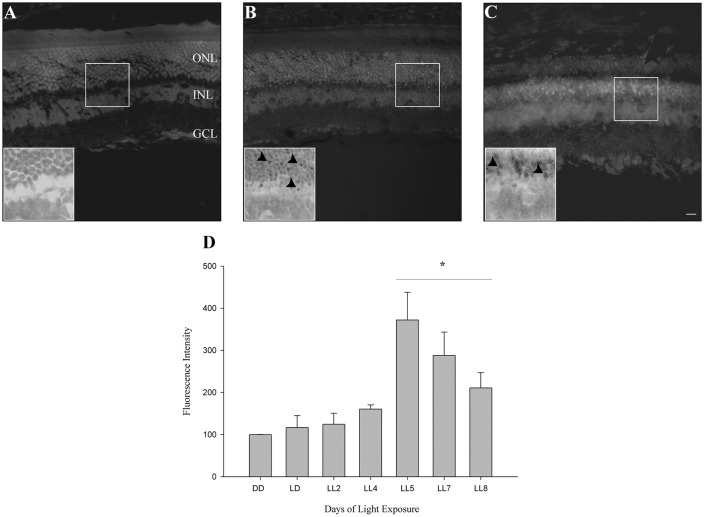
Oxidative stress study. **(A–C)** DHE staining at DD, LL2 and 8 days of light-emitting diode (LED) light exposure. Arrowheads show DHE positive cells in the ONL. **(D)** Reactive oxygen species (ROS) production detection by Flow cytometry using the DCFH-DA probe in controls (DD and LD) and in animals exposed to 2, 4, 5, 7 and 8 days to constant light (LL2–8). Data are mean ± SE, *n* = 1 animal/group from three independent experiments, *p < 0.05 vs. DD by Kruskal Wallis. ONL, outer nuclear layer; INL, inner nuclear layer; GCL, ganglion cell layer. Scale bar indicates 30 μm.

In order to know if retinas from animals exposed to constant light have oxidative stress, DHE was measured in retinal sections as indicated in “Materials and Methods” section. DHE and superoxide anion reaction produces ethidium (E), which binds to DNA rising the fluorescence in the cells (Gomes et al., 2005; Fernandes et al., 2006). Here, we show that retinas from animals exposed for 2 days of LL (LL2) have fluorescent label in few cells in ONL; however, at 8 days of LL (LL8) a significant increment of DHE label was observed (Figures 2A–C), correlating with ONL cell reduction (Figure 1). Besides the clear staining in ONL, no positive labeled in other retinal layer were observed in any time of LL studied, indicating the existence of oxidized products specifically in this layer.

#### Reactive Oxygen Species Quantification

To determine the kinetic of oxidative stress events occurred in ONL of retinas exposed to LL, ROS production was determined by flow cytometry using the DCFH-DA probe as indicated in “Materials and Methods” section. At 2 and 4 days of LL no significant increases in fluorescence were observed (124.70 ± 44.94 and 160.69 ± 16.9%, respectively). However, constant light treatment increases the levels of fluorescence, showing significant changes after 5 days of LL (LL5) where the production of ROS was maximal (372.16 ± 113.44%) respect to control in DD (100%). Although at 7 and 8 days of LL the fluorescence production decreased in correlation with cell death in the ONL (287.97 ± 221.55 and 211.00 ± 80.80%, respectively), ROS production was greater than in DD (Figure 2D).

#### Catalase Activity Determination

**Figure 3:**
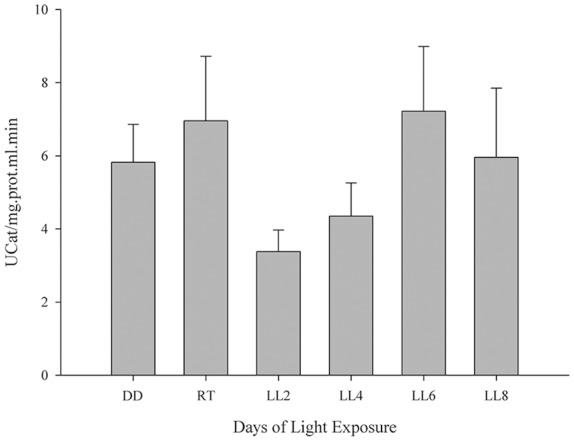
Catalase activity study in retinal light damage. CAT activity expressed as U/mg prot.ml.min in controls (DD and RT) and rats exposed at different days of LL. Data are mean ± SE, *n* = 1 animal/group from three independent experiments, *p* = 0.216 by one-way ANOVA and Duncan’s *post hoc* test.

CAT enzyme is known to contribute to H_2_O_2_ detoxification in the retina and the inhibition of its activity increases H_2_O_2_ concentration 2.5-fold, which cannot make up for the GPX activity (Ohta et al., 1996) the enzyme being an antioxidant factor in retina. In order to investigate the CAT activity and its association in the prevention of oxidative stress in constant light exposed rats, it was assessed as indicated in “Materials and Methods” section. Although constant light stimuli increased lightly CAT activity at 6 and 8 days of LL (7.22 ± 3.95 and 5.96 ± 3.27, respectively) with respect to LL 2 and 4 (3.38 ± 1.44 and 4.35 ± 2.24, respectively), reaching the levels of DD and RT (5.82 ± 2.55 and 6.96 ± 4.30, respectively) there were no significant differences in any times of LL measured (Figure 3), suggesting that CAT is affected by the desynchronization of animals by constant light exposure.

#### Retinal Fatty Acid

**Figure 4:**
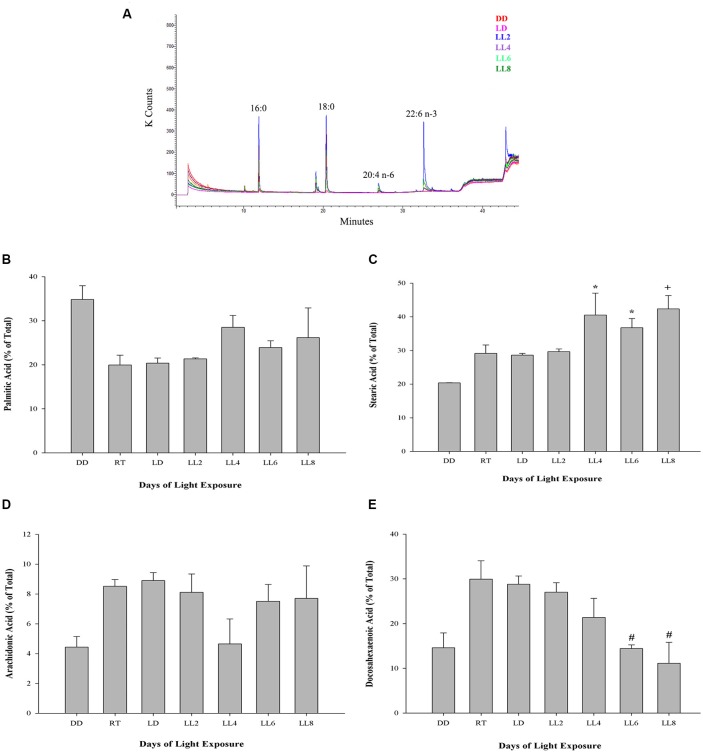
Fatty acid composition in light-treated rat retinas. **(A)** Full scan GC-MS total ion chromatograms of rat retina from controls (DD and LD) and rats exposed to 2, 4, 6 and 8 days of LL. 16:0, palmitic acid; 18:0, stearic acid; 20:4 n-6, arachidonic acid; 22:6 n-3, docosahexaenoic acid. **(B–E)** Quantification of palmitic **(B)**, stearic **(C)**, arachidonic **(D)** and docosahexaenoic acid **(E)** from controls (DD, RT and LD) and animals exposed for 2, 4, 6 and 8 days of constant light (LL), expressed as a percentage of total fatty acids, respectively. Data are mean ± SE, *n* = 1 animal/group from three independent experiments, *p < 0.05 vs. DD and LD; ^+^*p* < 0.05 vs. DD; ^#^*p* < 0.05 vs. RT and LD, by Kruskal-Wallis.

**Figure 5:**
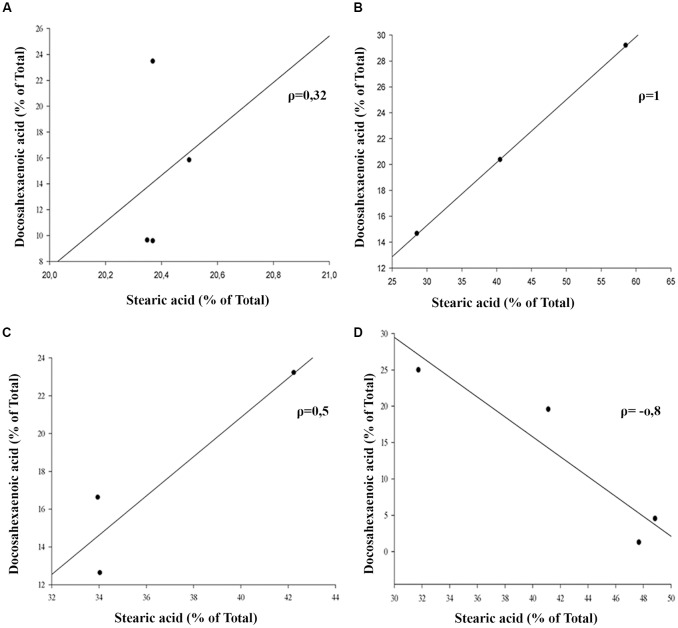
Linear correlation analysis between stearic and docosahexaenoic acid in light-treated retinas. **(A)** Control animals maintained in DD.** (B–D)** Animals maintained for 4, 6 and 8 days at constant light, respectively. ρ: pearson correlation coefficient, *n* = 1 animal/group from three independent experiments.

The outer segment membrane of retinal rods and cones contain high polyunsaturated fatty acids (PUFAs) where docosahexaenoic (22–6 n-3, DHA) and arachidonic acid (20:4 n-6, AA) are the major species present. Because PUFAs are a target for oxidation, the analysis by GC-MS in rat retinas from animal exposure to different LL allow us to determine if there are changes in the membrane components during RD as a result of light exposure. Individual fatty acids were identified comparing the mass spectra with those present in the NIST library and their retention time with a commercial standard (see “Materials and Methods”). Representative GC/MS analyses of fatty acids from retinas of control animals and exposed to light (LL) are shown in Figure 4A. Palmitic (16:0, PA) and arachidonic acid (20:4 n-6, AA), did not show statistical differences in any times of LL studied (Figures 4B,D). However, stearic acid (18:0, EA) shows increased levels with respect to control at LL4, maintaining this higher level at LL6 and LL8 (Figure 4C) in association with the DHA decrease which was significant at LL 4 and 8 (Figure 4E). In order to determine if the variations were related, we perform a correlation analysis between EA and DHA in control animals (DD) and animals exposed to 4, 6 and 8 days to LL. Figures 5A–D show no correlation in control animals maintained in dark (DD) and in LL6, however, there is a positive correlation in LL4 (*ρ* = 1) and negative correlation in LL8 (*ρ* = −0.8).

### Electroretinograms Responses

Previously we demonstrated retinal functional alterations in the scotopic ERG responses, showing that throughout the days of LL, the two principal waves “a” and “b” tend to decrease their amplitudes and increase their latency time, reaching abolished records after LL4, indicating that photoreceptor cells fail between the first 4 days of LL (Quintana et al., 2016). Here, to study the effects of light interruption by darkness, the animals were exposed to constant light with 2, 4, 6, 10 or 12 h of dark during the subjective night as indicated in “Materials and Methods” section. As shown in Figure 5, with 2, 4 and 6 h of rest in darkness per day during subjective night, the ERG show abolished records as we described before, however, at 8 and 10 h of dark the ERG began to restore, reaching normal values at 12 h. This result indicates that including hours of rest in darkness during the subjective night the effects of light exposure may be less harmful than constant stimuli.

## Discussion

The excess of artificial light exposure constitutes a problem that is emergent worldwide. The behavior of excessive exposure to light might have effects on the vision, promoting RD and circadian asynchrony in healthy population. We have previously characterized a RD model that may provide the possibility to study specific events associated with RD under conditions of low light with LED sources, at constant or long-term periods of exposure (LP context). We demonstrated that photoreceptor cells died along the days of continuous light stimuli through a caspase-3 independent mechanism. Rhodopsin analysis did not show a reduction in protein levels, however, rhodopsin-phospho-Ser334 increased gradually with the days of LL exposure indicating increasing levels in the phosphorylation. The study of rhodopsin allows us to investigate the role of phototransduction mechanism in the RD model suggesting that rhodopsin dysregulation could be involved in one of the ways of RD (Contín et al., 2013). Furthermore, in the inner retina, we demonstrated a relocation of non-visual photopigments (OPN4 and OPN5); however, inner retinal cells survival were not affected, indicating a compensatory mechanism of protection with possible changes in synchronization of circadian rhythms (Benedetto et al., 2017). To further describe the molecular pathways of cell death, in this work, we have examined the oxidative stress involvement in the effects of constant exposure to LED sources. Results from both DHE and DCFH-DA probe analysis demonstrate the existence of oxidative reactions in LL exposed rats (Figure 2). The ONL label of DHE indicates the occurrence of oxidative stress only in this layer, discarding stress mechanisms in other cells of the retina, coinciding with cell death study. DCFH-DA analysis shows that the production of ROS increases significantly after LL5 with higher levels at this day of LL (Figure 2D) indicating oxidative stress from 5 days of constant light. Powerful defenses against oxidative stress are the effect of Vitamin E, Ascorbic Acid, and melanin as well as the activity of antioxidant enzyme superoxide dismutase (SOD), CAT and GSHPx (Yusifov et al., 2000). Here, we demonstrated that CAT activity does not present significant differences in all times studied (Figure 3) showing a light diminution at LL2 to LL4 with respect to controls in DD and LD treatments; however, at LL6 and LL8 the CAT activity increases showing levels similar to the controls. Previous studies have demonstrated that CAT enzyme is finely regulated by the circadian rhythms; CAT bioinformatic and mRNA studies have been shown putative E-box sites in CAT and GPx regulatory regions; furthermore, CAT mRNA expression and enzyme activity shows circadian rhythmicity with higher levels at the end of the day (Navigatore Fonzo et al., 2009; Kharwar and Haldar, 2012; Lacoste et al., 2017). Sani et al. (2006) have demonstrated that CAT activity shows changes across a 24-h period in mouse. Thus, we hypothesize that constant light might produce a complex and multifactorial biological process inducing progressive desynchronization of circadian rhythm with concomitant antioxidant function altered; and therefore, the break of the equilibrium between ROS over-production and antioxidant processes. However, further studies are necessary in order to conclude an association between them.

The vertebrate retina contains high concentration of PUFA; particularly, in the outer segment membrane of retinal rod and cones, the PUFAs DHA and AA are the major species examined (Fliesler and Anderson, 1983; Giusto et al., 2000; SanGiovanni and Chew, 2005). PUFAs are essential for maintaining the appropriate fluidity of the membrane, necessary for efficient phototransduction cascade (Brown, 1994; Gawrisch and Soubias, 2008). The composition of membrane lipids and their direct interaction with proteins play an important role in the modulation of the rhodopsin function (Salas-Estrada et al., 2018). A good retinal function is subject to adequate membrane structure which contains high levels of DHA. Anderson and Penn (2004) proposed, as neuroprotective adaptive responses, changes in DHA levels at different environmental conditions with the aim to control the number of photons captured by rhodopsin molecule. In the fatty acid n-3-deficient rod outer segment, it has been shown that reduced rhodopsin transducin (Gt) coupling, reduced cGMP phosphodiesterase activity, and slower formation of metarhodopsin II-Gt complex, relative to the animals fed with n-3-adequate diet, explained the reduced activity of rod phototransduction in these animals (Anderson and Penn, 2004). These findings support the so called “photostasis” which infers the morphological and biochemistry adaptation to capture a constant number of photons. The constant light exposure may break the adaptive mechanism and promote several pathways of cell death.

**Figure 6:**
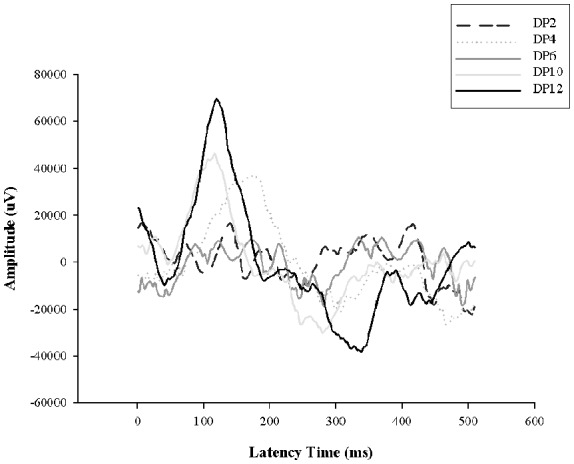
Electroretinogram (ERG) analysis in animals exposed to dark period. Animals exposed for 8 days of constant light with dark periods of 2, 4, 6, 10 and 12 h per day during subjective night (DP 2–8); DD, constant dark. DP, dark period.

DHA and AA are obtained from two ways: (a) the diet or (b) synthesized from alpha-linolenic (18:3n3) and linoleic acids (18:2n6). DHA is transported from the liver to the RPE cells (Scott and Bazan, 1989). In rat rods, DHA is essential for normal development of function (Benolken et al., 1973; Wheeler et al., 1975) where rat deficient in PUFA showed reduced ERG responses (Jeffrey et al., 2002). DHA located mainly in photoreceptors represents 50% of the total fatty acid and recently it has been demonstrated that the membrane of rod outer segment has higher levels of PUFA and Very Long Chain-PUFA (VLC-PUFA) in rod-dominant rats than cone-dominant, suggesting that rods and cones do not have equal lipid requirements (Agbaga et al., 2018). The cell membranes are susceptible to oxidation due to high level of unsaturation of PUFAs, furthermore, retina membranes exposed to light, elevate the concentration of oxygen with the presence of rhodopsin photo-bleached products that increment the risk (Kagan et al., 1981; Wiegand et al., 1983; Rózanowska and Sarna, 2005; Hunter et al., 2012). Earlier study revealed that lipid peroxidation reactions in light-induced RD induce the specific loss of DHA from rod outer segment membranes during constant illumination with an increase in the production of lipid hydroperoxides. None of the other fatty acids, including the AA, changed significantly over the 3-day time periods; concluding that light mediates the peroxidation of PUFAS in outer segment membrane acids and supporting the hypothesis that peroxidation is involved in retinal light degeneration (Wiegand et al., 1983). So, as we exposed the rat retinas to constant light at 200 lux, and we demonstrated increase in ROS significantly at LL5, we decided to study if fatty acid composition is altered belonging to the days in LL, specially by oxidative stress processes. Results from GC-MS analysis shows that PA (saturated) and AA (unsaturated) do not show statistical changes with the days of LL. However, DHA (unsaturated) decreased with the LL exposure with statistical significance at LL4, 6 and 8 compared with controls in LD and RT (Figure 4E). Remarkably, the decrease of DHA draws a parallel with an SA increase at the same day of LL (Figure 4C). The study of the correlation between both fatty acids shows an association between them in LL-treated retinas; instead, we did not find any association in control animals (DD). We assumed that the reduction in DHA may be affected by the oxidative stress in photoreceptor outer segment which in turn affects the photoreceptor survival through unknown different miss-regulation mechanism involved. The hepatic metabolism, through desaturation and elongation steps, influences the fatty acid composition (Engler et al., 2000). Diabetes, atherosclerosis, neurological disorders, cancer and others are enhanced by the disturbances in fatty acid content (see Zolfaghari and Ross, 2003). One fatty acid biosynthesis pathway begins with the desaturation of PA and SA; in this way, delta-9-desaturase (Δ9-desaturase) is the key enzyme necessary for the conversion from palmitic to palmitoleic acid (16:1n-7) and stearic to oleic acid (18:1n-9). Δ6 and Δ5-desaturase enzymes are required for the metabolism of essential fatty acids, linoleic (18:2n-6) and alpha-linolenic (18:3n-3), to long-chain PUFAs (LCP-DHA). Hormonal, dietary factors, peroxisomal proliferators and developmental processes can affect the activity of Δ9-desaturase, which alters monounsaturated and PUFA composition (Ntambi, 1995, 1999). In hypertensive rats, DHA-fed Δ9-desaturase activity decreases at 53% suggesting that dietary DHA influences properties and function of cellular membranes due to changes in fatty acid composition (Engler et al., 2000). Animals with Δ9-desaturase activity dysregulation show changes in the relation of stearic:oleic acid with effects over membrane fluidity and function (Ntambi, 1995, 1999). Lai et al. (2010) saw that patients with BCD (Bietti crystalline dystrophy; a retinal degenerative disease) have higher concentration of stearic acid and lower octadecadienoic acid (18:1n-9) than healthy animals. Furthermore, the activity of Δ9-desaturase and the concentration of monounsaturated fatty acids were lower in BCD animals suggesting abnormalities in lipid metabolism demonstrating a direct relation between rising levels of stearic acid, lowering fatty acid concentration and retinal dysfunction (Lai et al., 2010). We think that reduction of DHA levels by oxidation in LL rats may affect the desaturase activity in retina causing the rising of stearic acid accumulation which might affect the membrane proprieties giving more rigidity to the outer segment. It may explain part of the cell death mechanism induced by oxidative stress. Furthermore, the excess of light may alter the levels of Neuroprotectin D1 (NPD1) due to the DHA oxidation. NPD1 is a biologically active DHA derivative which induces downstream pro-survival pathways such as gene expression, pro-apoptotic gene suppression and pro-inflammatory responses, among others. The diminution of DHA may alter these ways across inducing photoreceptor cell death (see Asatryan and Bazan, 2017). Other studies are required to better understand the effects of DHA reduction; nevertheless, analyzing the time course of oxidative stress, we consider that it is not the only pathway involved, because the production of DHE and ROS have a maximum expression after LL5-6 (Figure 2), and the reduction of ONL occurs between LL6 and LL8; however, the two principal waves of ERG, a and b, tend to decrease the amplitudes and increase the latency time during light stimuli, reaching abolished records at LL4 (Quintana et al., 2016). All these results suggest that a dysfunction of retinal electrical activity occurs previously to redox imbalance. It has been demonstrated that low intensities of light stimuli need the activation of photopigments and phototransduction to induce RD (Hao et al., 2002; Grimm and Remé, 2013), suggesting that impairment of the phototransduction mechanism could be responsible for cell death. Besides, previously we demonstrated the existence of more phosphorylated rhodopsin (rhodopsin-phospho-Ser^334^) after LL2 with a higher level at LL7, supporting the idea that changes in phototransduction cascade are also involved (Contín et al., 2013). Here, we show scotopic ERG responses abolished until 6 h of rest darkness during subjective night; however, after 8 h of dark the activity begin to restore, reaching normal values at 12 h (Figure 6). These results suggest that the existence of regulatory mechanisms tend to revert or prevent the process of cell death when the retina is maintained in rest (notice the difference between 10 and 12 h of darkness); nevertheless, when light exposure is prolonged a threshold is exceeded promoting a chain of different cell death pathways. Further studies are necessary to determine the role of opsin-mediated RD processes in this model; however, we think that retinal dysfunction during the 1st days of LL (LL1-LL4) is promoted by phototransduction processes which may induce other pathways of cell death as the oxidative stress occurr. Therefore, prevention therapies with antioxidants would not completely solve RD by LED light.

## Ethics Statement

Comité Institucional de Cuidado y Uso de Animales de Laboratorio en el ámbito de la Facultad de Ciencias Químicas—CICUAL-FCQ-en los proyectos científicos -fs. 6-EXP-UNC:0007526/201 8 Number 740.

## Author Contributions

MB has contributed with the hypothesis, has performed the experiments, the analysis of the results and discussion. MC is the corresponding author, and had written the manuscript.

## Conflict of Interest Statement

The authors declare that the research was conducted in the absence of any commercial or financial relationships that could be construed as a potential conflict of interest.
